# Coxsackievirus A24 variant associated with acute hemorrhagic conjunctivitis outbreak in Bhutan, 2023

**DOI:** 10.1038/s41598-025-94776-7

**Published:** 2025-04-28

**Authors:** Sonam Gyeltshen, Tshering Dorji, Kunzang Dorji, Sonam Wangchuk, Dorji Tshering, Yongyuth Poolpanichupatam, Piyawan Chinnawirotpisan, Wudtichai Manasatienkij, Kathryn A. McGuckin Wuertz, Aaron R. Farmer, Chonticha Klungthong

**Affiliations:** 1 Royal Centre for Disease Control, Ministry of Health, Royal Government of Bhutan, Thimphu, Bhutan; 2 Phuntsholing General Hospital, National Medical Services, Royal Government of Bhutan, Phuntsholing, Bhutan; 3 Department of Virology, Walter Reed Army Institute of Research-Armed Forces Research Institute of Medical Sciences, Bangkok, Thailand

**Keywords:** Acute Hemorrhagic Conjunctivitis, Pink eye, Coxsackievirus A24 variant, NGS, Outbreak Bhutan, Virology, Metagenomics, Viral epidemiology, Phylogeny, Viral infection

## Abstract

In 2023, Bhutan detected an outbreak of acute hemorrhagic conjunctivitis (AHC) in southern and central regions, coinciding with similar outbreaks reported in South and Southeast Asia. Laboratory results from clinical specimens were initially inconclusive in identifying the etiological agent. To address this, 18 clinical samples, comprising conjunctival swabs and throat/nasal swabs from nine patients were collected and sent to WRAIR-AFRIMS for further analysis. Specimens were tested using multiplex real-time RT-PCR (Fast-track respiratory 21 kit, FTD21) and hybrid-capture-based next-generation sequencing (NGS) with the Illumina Viral Surveillance Panel. FTD21 testing identified human adenovirus, human bocavirus, influenza A, enterovirus, and/or human rhinovirus in 10/18 specimens (56%). A higher detection rate was observed in conjunctival specimens (78%, 7/9) compared to throat/nasal specimens (33%, 3/9) from the same patients, highlighting the increased sensitivity of conjunctival samples in identifying causative agents of conjunctivitis. Further assessment by NGS detected only coxsackievirus A24 variant (CVA24v) genotype IV in 9/17 specimens (53%), with detection primarily from conjunctival samples. Phylogenetic analyses of CVA24v VP1 sequences revealed genetic distinctions in the 2023 isolates compared to prior outbreaks from 2002–2017, suggesting re-emergence driven by novel genetic mutations. These findings suggest that conjunctival samples are more reliable for detecting the etiological agent in AHC outbreaks compared to throat/nasal swabs. Additionally, the identification of a novel strain of CVA24v genotype IV underscores the importance of genomics.

## Introduction

Acute Hemorrhagic Conjunctivitis (AHC), or pink eye, is a common ocular condition affecting individuals across various age groups and socioeconomic strata^1^. AHC is typically a self-limiting infection, manifesting within 2 days of infection, with sudden onset of painful, swollen, red eyes with subconjunctival hemorrhages, excessive tearing, and preauricular lymphadenopathy noted in some AHC patients^2^. The two primary etiological agents of AHC are Coxsackievirus A24 variant (CVA24v) and Enterovirus 70 (EV70), although several other enteroviruses, including human enteroviruses 71 (EV71) and 68 (EV68), as well as human adenoviruses (HAdv), are also known to cause the disease^2,3^. The first documented cases of AHC occurred in Ghana in 1969, where EV70 was identified as the etiological agent^4^. The following year, an outbreak of AHC occurred in Singapore, caused by CVA24v, which was initially confined to Southeast Asia^5,6^. Since then, CVA24v has spread to other regions, causing notable outbreaks in Africa, Europe, North America, and South America. AHC has now become a global public health concern, with more than ten million reported cases worldwide^7–9^.

Following its initial detection in 1970^5^, CVA24v has consistently emerged as the predominant etiological agent in AHC outbreaks^9^. CVA24v belongs to the genus *Enterovirus* under the family *Picornaviridae*, which has a positive-sense, single-stranded RNA genome of about 7.4 kilobases^10^. The genome contains an extended open reading frame (ORF) that encodes a polyprotein, consisting of four structural capsid proteins (VP1-VP4) and seven non-structural proteins (2A-2C and 3A-3D) involved in viral replication and virus-host interaction^11^. Genetic diversity in the VP1 region has been used to classify CVA24v into eight genotypes (GI–GVIII)^8^. The earliest reported genotype, GI, was identified in Singapore in 1970 ^5^, followed by GII and GIII, which circulated widely and caused outbreaks in Asia and Africa during the 1980s and 1990s. Genotype GIV, first detected in the early 2000s^12^, rapidly replaced GII and GIII as the dominant lineage globally. Since its emergence, GIV has been associated with large-scale outbreaks across Africa, Asia, and the Americas, including major epidemics in Brazil and other South American countries in the early 2000s. It remains the primary driver of recent outbreaks in South and Southeast Asia^7–9,12^.

In recent decades, South and Southeast Asia have reported numerous outbreaks of AHC, characterized by its sudden onset and rapid spread. These outbreaks were typically localized and self-limiting, resolving without significant spread across the broader population^13,14^. However, in 2023, AHC outbreaks were documented in multiple countries, including Vietnam, India, Ghana, and Pakistan, with CVA24v identified as the primary pathogen in India and Pakistan^15,16^. Concurrently, Bhutan encountered a significant upsurge in conjunctivitis cases, with nearly 10,264 reported cases between June to October 2023, potentially aggravated by the reopening of schools after summer break in the affected districts^17,18^. AHC presents with a sudden onset of conjunctival hyperemia, ocular pain, and subconjunctival hemorrhages^1^. While clinical assessment remains the cornerstone of AHC diagnosis, laboratory investigations are employed to identify the underlying etiology and support differential diagnosis. In Bhutan, the diagnostic capacity for identifying the causative agents of conjunctivitis outbreaks has been historically limited. During the 2023 outbreak, the Royal Centre for Disease Control (RCDC) sought assistance from the Walter Reed Army Institute of Research-Armed Forces Research Institute of Medical Sciences (WRAIR-AFRIMS) in Bangkok, Thailand, to investigate the outbreak.

This study investigated the 2023 conjunctivitis outbreak in Bhutan by analyzing 18 clinical specimens, comprising 9 conjunctival swabs and 9 throat/nasal swabs collected from the same patients, using a combination of multiplex real-time RT-PCR and next-generation sequencing (NGS). By leveraging advanced pathogen detection technologies, the study aimed to identify etiologic agents and provide critical insights to inform future public health responses.

## Results

### Epidemiological findings of the outbreaks

**Fig. 1:**
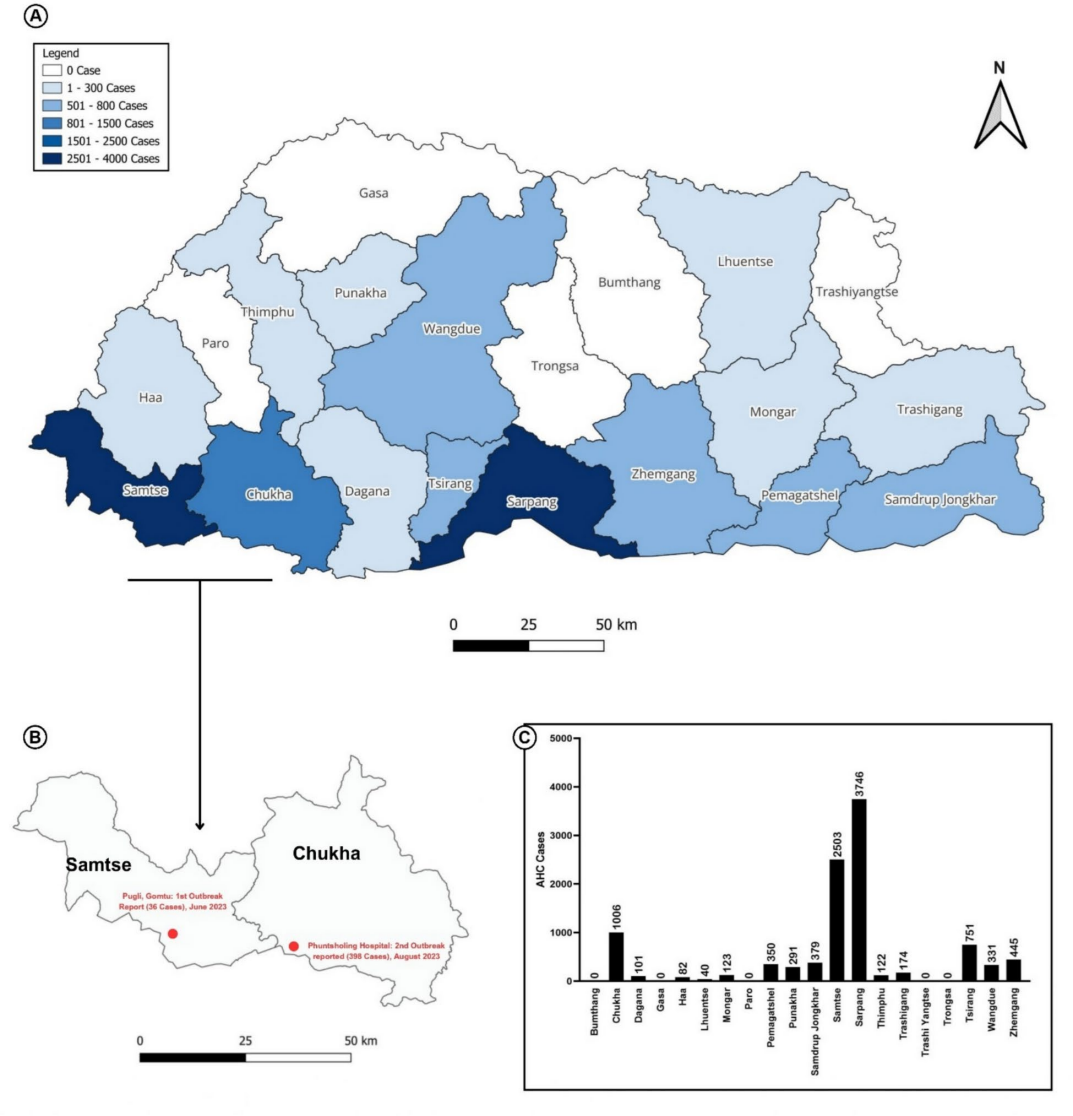
Distribution of AHC cases reported across districts in Bhutan through event-based surveillance (NEWARS), 2023. This figure shows the distribution of AHC cases recorded in Bhutan between June and October 2023. **a**) Map of Bhutan showing the total number of AHC cases reported by each district. **b**) Highlighted inset maps of Samtse and Chukha districts, showing the first localized outbreak in Samtse and the larger-scale outbreak in Chukha that resulted in a substantial case burden. **c**) Bar graph showing the total number of AHC cases reported by each district. The map was created with qgis version 3.40.2 (https://www.qgis.org/).

**Fig. 2:**
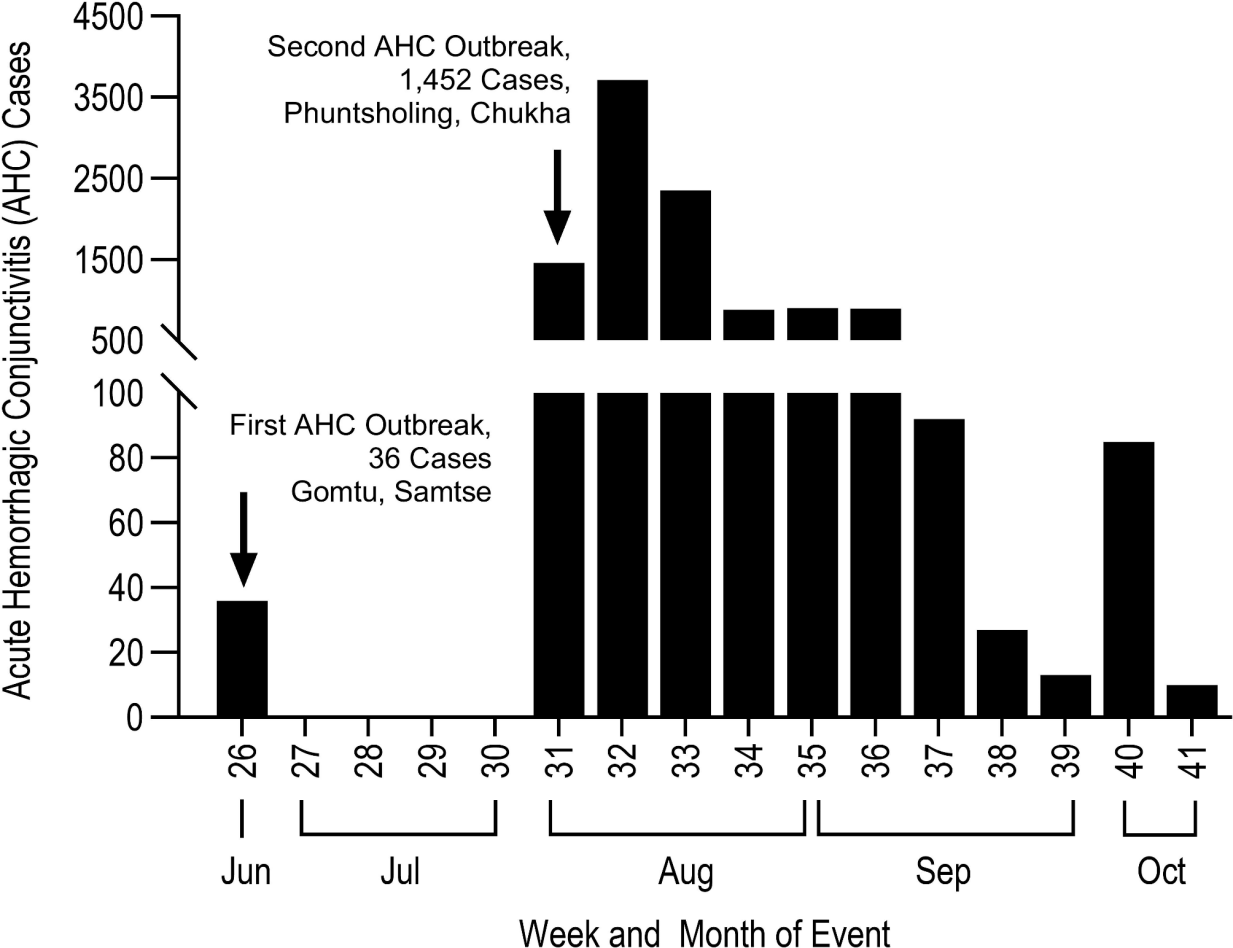
AHC cases reported to NEWARS from June 2023 to October 2023.

Between June and October 2023, Bhutan experienced 60 AHC outbreaks (defined by any occurrence of any illness in a community more than normal expectancy or unusual illness per National Early Warning Alert and Response Surveillance (NEWARS) guideline) resulting in a total of 10,444 reported cases across 15 districts of 20 districts in the country **(**Fig. 1**)**. Sarpang district reported the highest number of cases (3,746; 35.86%), followed by Samtse (2,503; 23.9%) and Chukha (1,006; 9.6%). Lhuntse and Haa districts reported the fewest cases, with 40 and 82 cases, respectively (Fig. 1). The 2023 outbreak was first reported in Pugli, a small town bordering India in Samtse, Bhutan, with 36 cases reported on June 20, 2023, through the National Early Warning, Alert and Response Surveillance Information System (NEWARSIS). This outbreak subsided over time without further public health interventions. However, in the later months there was a significant surge in AHC cases reported in Phuntsholing. A total of 398 cases from six schools were reported by Phuntsholing Hospital on 1^st^ August. Subsequently, numerous outbreaks occurred nationwide, with the highest number of cases reported on August 10^th^, reaching 2,674 cases in a single day. The cases were monitored by the RCDC through NEWARSIS. A timeline for daily cases was constructed to track the temporal distribution of AHC cases **(**Fig. 2**)**.

The outbreaks were isolated and irregular, occurring intermittently and geographically dispersed. The number of cases declined gradually over time, and no cases were reported after 9 October 2023.

The most common symptoms were ocular pruritus and conjunctival hyperemia, which were frequently accompanied by sensations of discomfort or the presence of a foreign body. Other commonly reported ocular signs included photophobia, eyelid edema, and excessive lacrimation. A subset of patients also presented with rhinorrhea, cough, and headache. Clinical examination showed conjunctival congestion as the primary finding, while blepharitis and conjunctival chemosis were observed less frequently. Symptom data from outbreak sites were collected at the cohort or group level rather than on an individual patient basis, limiting the availability of case-specific details. Management strategies primarily involved supportive therapies, including the use of cold compresses, dark glasses, and strict hand hygiene. For severe cases, antibiotic eye drops were prescribed by primary care providers. Notably, no long-term complications were noted.

### Detection and identification of pathogen

A total of 18 specimens (9 conjunctival and 9 throat/nasal specimens) were collected from the outbreak and sent for laboratory testing at WRAIR-AFRIMS. All 18 collected specimens were tested by multiplex real-time PCR. The results from FTD21 multiplex real-time RT-PCR are presented in Table 1. The results showed that human adenovirus (HAdV), human bocavirus (HBoV), human rhinovirus (HRV), enterovirus (EV), and influenza virus A (Flu A) were detected in 10 out of 18 specimens (7 conjunctival and 3 throat/nasal specimens) with the breakdown as follows: HAdV (3), HAdV and HBoV (1), EV and HRV (5), and Flu A (1). Across specimen type, 78% (7/9) of conjunctival specimens and 33% (3/9) of throat/nasal specimens tested positive by FTD21.

**Table 1:** Results of respiratory viral pathogen detection by multiplex real-time RT-PCR.

Patient	Collection Date	Age	Gender	Collection Site	Specimen No	Specimen Type	Multiplex real-time RT-PCR Results	
							Results^1^	Ct values^2^
Patient #1	17-Aug-23	13	Female	Phuntsholing	RCDC-001C	Conjunctival Swab	EV and HRV	EV; 33.65, HRV; 34.39
					RCDC-010 T	Throat/Nasal Swab	NEG	Undetermined
Patient #2	17-Aug-23	23	Female	Phuntsholing	RCDC-002C	Conjunctival Swab	HAdV	34.25
					RCDC-011 T	Throat/Nasal Swab	NEG	Undetermined
Patient #3	17-Aug-23	33	Female	Phuntsholing	RCDC-003C	Conjunctival Swab	HAdV	33.66
					RCDC-012 T	Throat/Nasal Swab	EV and HRV	EV; 34.07, HRV; 34.49
Patient #4	17-Aug-23	38	Male	Phuntsholing	RCDC-004C	Conjunctival Swab	EV and HRV	EV; 30.26, HRV; 32.47
					RCDC-013 T	Throat/Nasal Swab	NEG	Undetermined
Patient #5	17-Aug-23	70	Female	Phuntsholing	RCDC-005C	Conjunctival Swab	EV and HRV	EV; 30.69, HRV; 32.24
					RCDC-014 T	Throat/Nasal Swab	HAdV and HBoV	HAdV; 34.40, HBoV; 35.46
Patient #6	17-Aug-23	63	Male	Phuntsholing	RCDC-006C	Conjunctival Swab	NEG	Undetermined
					RCDC-015 T	Throat/Nasal Swab	Flu A	30.09
Patient #7	17-Aug-23	34	Male	Phuntsholing	RCDC-007C	Conjunctival Swab	HAdV	34.34
					RCDC-016 T	Throat/Nasal Swab	NEG	Undetermined
Patient #8	17-Aug-23	30	Male	Phuntsholing	RCDC-008C	Conjunctival Swab	EV and HRV	EV; 34.11, HRV; 35.17
					RCDC-017 T	Throat/Nasal Swab	NEG	Undetermined
Patient #9	17-Aug-23	36	Male	Phuntsholing	RCDC-009C	Conjunctival Swab	NEG	Undetermined
					RCDC-018 T	Throat/Nasal Swab	NEG	Undetermined

Definitions: Undetermined, below detection limit; NEG, negative.

^1^If HRV and EV are positive, the patient has either a co-infection caused by both HRV and EV or a single EV infection.

^2^The cyclic threshold (Ct) values of the internal controls (ICs) for the tested samples ranged from 25.87 to 27.65, demonstrating that the extraction process was carried out effectively. Additionally, the Ct values of the positive controls for specific viruses ranged from 23.37 to 26.34, confirming that the amplification step was performed effectively and that no interfering materials impacted the process.

For next generation sequencing (NGS), only 17 collected specimens were analyzed, as one sample had an inadequate volume. The results from NGS are presented in Table 2. The total number of raw paired-end reads obtained from the tested samples ranged from 112,372 to 6,895,138 reads. The percentage of paired-end reads mapped to the CVA24v reference sequence (GenBank accession number KR78685.1) ranged from 0.01 to 40.85%. The CVA24v genome sequence was identified in 9 out of the 17 specimens (6 conjunctival and 3 throat/nasal specimens). The breadth of coverage ranged from 80.00% to 99.95%, and the median depth of coverage ranged from 26 to 9,763. No genome sequences of other pathogens were detected by NGS. For each specimen type, 67% (6/9) of conjunctival specimens and 33% (3/9) of throat/nasal specimens were tested positive for CVA24v by NGS. Among the results from the 17 samples, there were 6 discordant results between FTD21 and NGS. These included: 1) two samples that tested negative by FTD21 but positive for CVA24v by NGS, 2) one sample that tested positive for Flu A by FTD21 but was undetermined by NGS, 3) one sample that tested positive for HAdV by FTD21 but positive for CVA24v by NGS, and 4) one sample that tested positive for HAdV and HBoV by FTD21 but positive for CVA24v by NGS.

**Table 2:** Results of respiratory viral pathogen detection by NGS.

Patient	Specimen No	Specimen Type	NGS Results							
			Results	Total raw paired-end reads	Total trimmed paired-end reads	No. of paired-end reads mapped to the reference sequence	% Paired-end reads mapped to the reference sequence	% Breadth of coverage (≥ 10x)**	Median depth of coverage	Consensus sequence length (bp)
Patient #1	RCDC-001C	Conjunctival Swab	CVA24v	2,014,440	1,040,379	167,943	16.14	99.79	2,873	7,436
	RCDC-010 T	Throat/Nasal Swab	Undetermined	119,316	43,902	102	0.23	N/A	N/A	N/A
Patient #2	RCDC-002C	Conjunctival Swab	CVA24v	837,494	356,038	1,902	0.53	84.96	42	7,399
	RCDC-011 T	Throat/Nasal Swab	Undetermined	3,246,778	1,988,680	72	0	N/A	N/A	N/A
Patient #3	RCDC-003C*	Conjunctival Swab	N/A	N/A	N/A	N/A	N/A	N/A	N/A	N/A
	RCDC-012 T	Throat/Nasal Swab	CVA24v	1,710,760	697,978	27,202	3.9	99.51	628	7,447
Patient #4	RCDC-004C	Conjunctival Swab	CVA24v	4,312,848	2,556,531	36,165	1.41	99.48	703	7,401
	RCDC-013 T	Throat/Nasal Swab	Undetermined	112,372	29,636	0	0	N/A	N/A	N/A
Patient #5	RCDC-005C	Conjunctival Swab	CVA24v	1,972,464	1,214,088	495,903	40.85	99.95	9,763	7,436
	RCDC-014 T	Throat/Nasal Swab	CVA24v	6,895,138	2,953,147	16,438	0.56	99.38	381	7,441
Patient #6	RCDC-006C	Conjunctival Swab	Undetermined	6,454,776	2,918,808	166	0.01	N/A	N/A	N/A
	RCDC-015 T	Throat/Nasal Swab	Undetermined	938,972	500,838	0	0	N/A	N/A	N/A
Patient #7	RCDC-007C	Conjunctival Swab	Undetermined	1,148,374	482,708	134	0.03	N/A	N/A	N/A
	RCDC-016 T	Throat/Nasal Swab	Undetermined	1,797,024	806,435	126	0.02	N/A	N/A	N/A
Patient #8	RCDC-008C	Conjunctival Swab	CVA24v	376,776	206,279	16,413	7.96	99.5	326	7,434
	RCDC-017 T	Throat/Nasal Swab	Undetermined	445,502	215,660	0	0	N/A	N/A	N/A
Patient #9	RCDC-009C	Conjunctival Swab	CVA24v	144,804	39,937	6,209	15.55	99.19	103	7,387
	RCDC-018 T	Throat/Nasal Swab	CVA24v	2,920,248	1,636,539	1,097	0.07	80	26	7,326

Definitions: Undetermined, below detection limit; NEG, negative; N/A, not applicable.

*The sample had an inadequate volume for testing.

**The breadth of coverage is calculated based on the percentage of genome bases that have been sequenced with a depth of coverage of at least 10x, covering the entire length of the reference sequence. The reference sequence is a CVA24v complete genome sequence with GenBank accession number KR478685.1, which has a length of 7,439 bp.

### Viral genome characterization

Among the CVA24v genome sequences detected, eight complete genome sequences and one partial genome sequence were obtained and submitted to GenBank database under accession number PP505845-PP505853. BLAST search results indicated that these sequences were similar to CVA24v complete genome sequences available in GenBank during the time of the report, including sequences from India, Pakistan, and China collected between May to September 2023 (percent identity > 99% and E value = 0.0). All new CVA24v sequences analyzed in this study were identified as CVA24v genotype IV using version 2.13 of the Genome Detective Enterovirus Genotyping Tool (https://www.genomedetective.com/app/typingtool/etv/).

**Fig. 3:**
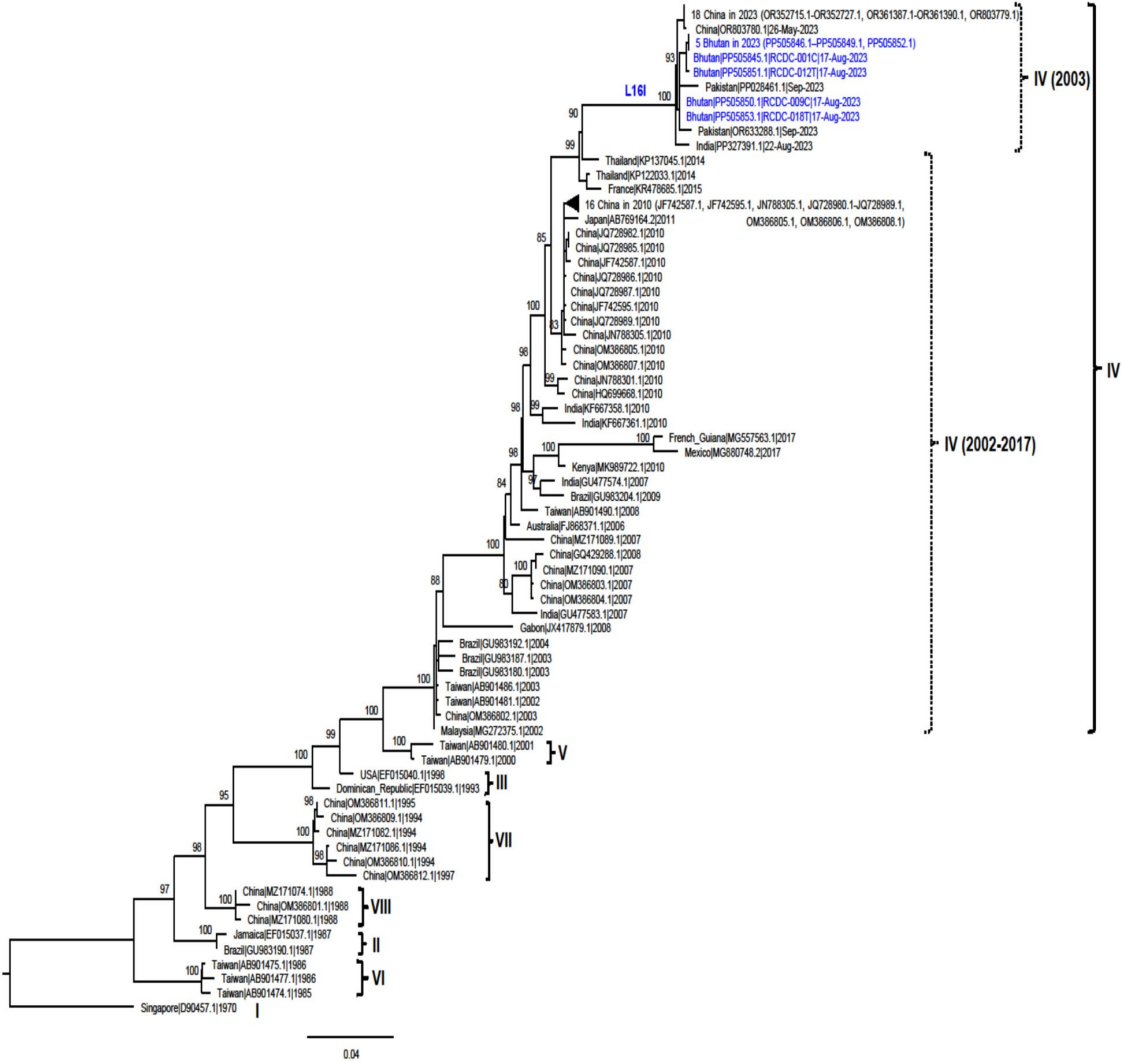
The Maximum likelihood tree of 96 VP1 gene sequences of CVA24v (915 nt), including 9 sequences were obtained from this study (blue), and 87 sequences were from GenBank (black). The tree was built using TNe + G4 substitution model. Bootstrap values greater than 80 are shown. Genotypes I to VIII are labelled. Amino acid substitution that distinguishes viruses from 2023-Genotype IV from old viruses in the same genotype is indicated.

**Fig. 4:**
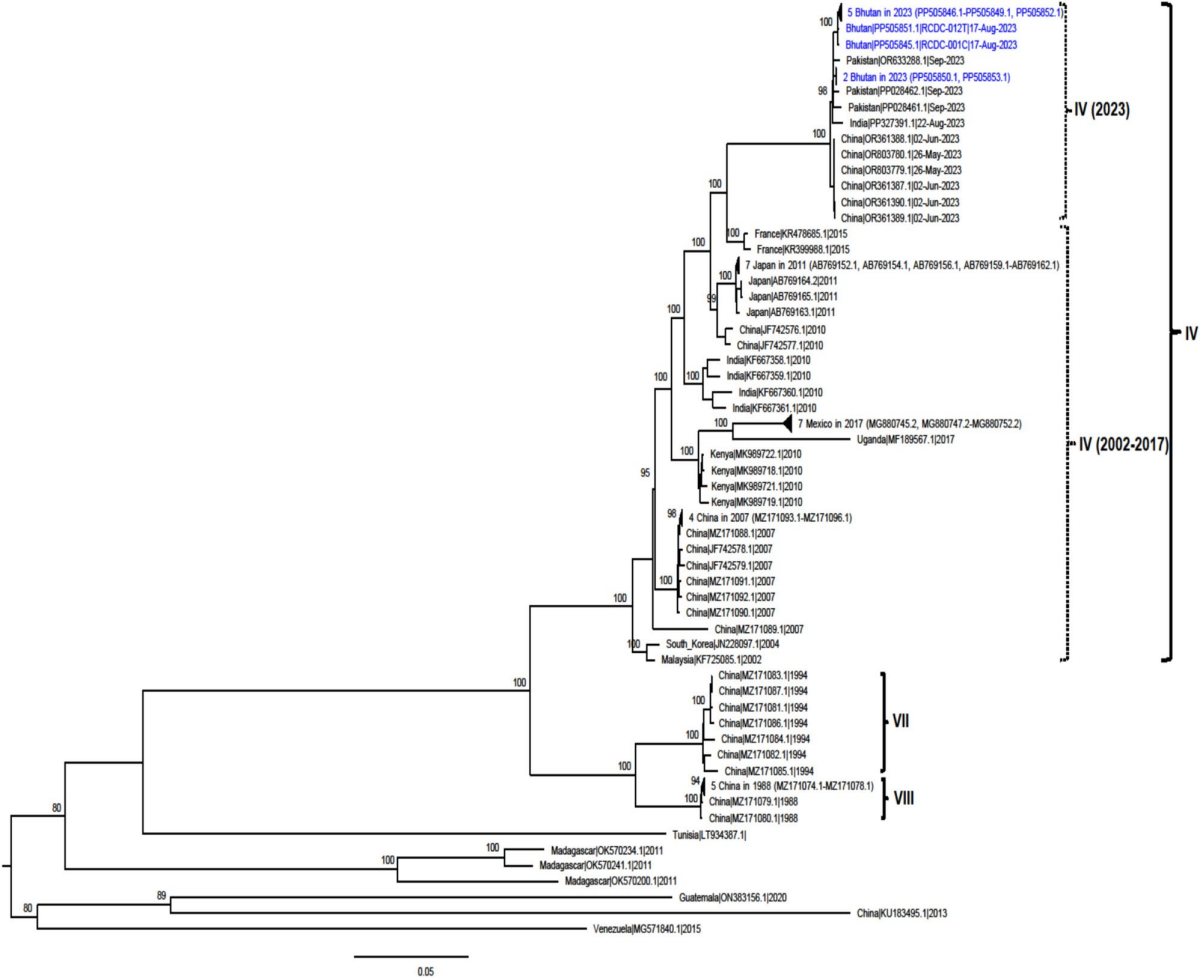
Maximum likelihood tree of 83 genome sequences of CVA24v (6,645 nt), including 9 sequences were obtained from this study (blue), and 74 sequences were from GenBank (black). The tree was built using GTR + F + I + G4 substitution model. Bootstrap values greater than 80 are shown. Genotypes IV, VII, and VIII are labelled.

**Fig. 5:**
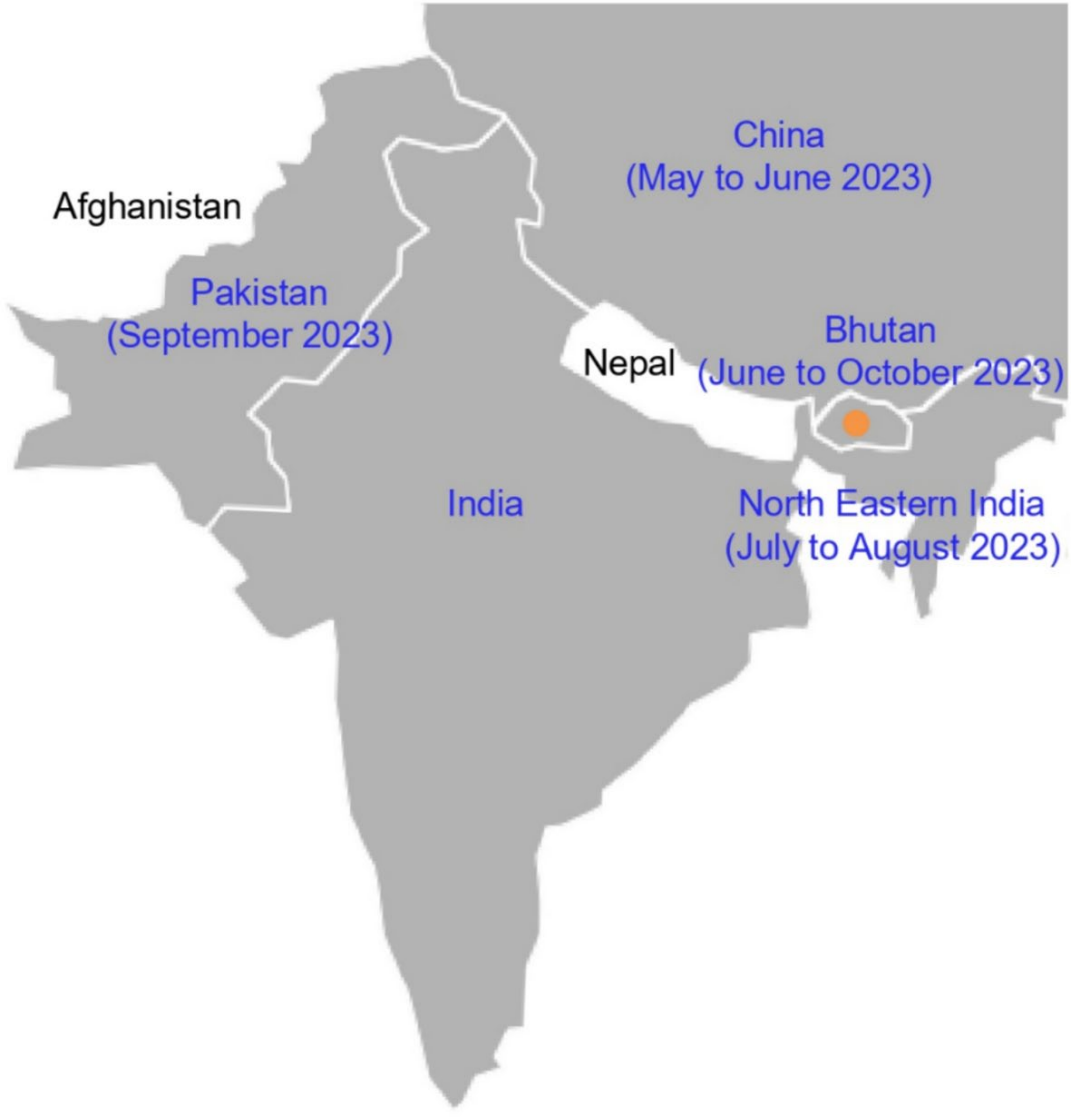
The map of countries from which CVA24v sequences were obtained in 2023 and submitted to GenBank, along with the time period of reports of conjunctivitis cases in each country. Outbreak data from other countries sourced from GenBank submissions, November 2023. This map was created using Python (version 3.9.15) with the geopandas library (version 0.14.4)^19^ for geographic processing and the matplotlib library (version 3.7.1)^20^ for plotting.

The maximum likelihood trees of the VP1 gene and the coding sequences of CVA24v are shown in Figs. 3 and 4, respectively. The trees reveal that all sequences obtained in this study clustered together with recent CVA24v sequences from China (collected May–June 2023), India (collected July–August 2023), and Pakistan (collected September 2023), forming a distinct group. This clustering indicates the emergence of a new lineage among CVA24v genotype IV strains circulating in 2023, supported by a bootstrap value of 100. These 2023 sequences are clearly separated from genotype IV isolates from prior years (2002–2017). The identification of this new lineage highlights the genetic divergence of CVA24v in 2023. Figure 5 shows the map of countries from which CVA24v sequences were obtained in 2023 and submitted to GenBank, along with the time period of reports of conjunctivitis cases in each country.

When comparing complete VP1 gene sequences of genotype IV from Bhutan with that of genotype IV from other countries in previous years (2002–2017) (n = 28) and 2023 (n = 22), the nucleotide identity ranged from 89.18 to 95.30% and 98.67 to 99.56%, respectively, while nucleotide divergence ranged from 4.70 to 10.82 and 0.44 to 1.33, respectively (Table 3). A total of 19 amino acid substitutions, unique to the CVA24v genotype IV collected in 2023 sequences and not observed in sequences of the same genotype from previous year (2002–2027), were identified. These included substitutions on proteins VP1 (L16I), VP2 (V146I and E166D), 2A (S44N, S57N, F/Y104H, and N117T), 2B (S30N), 2C (Q267R), 3A (V51I), 3B (T18A), 3C (S21N, S66T, and V75I), and 3D (P48A, M123L, E227D, T365K, and K395Q). These novel substitutions distinguish the 2023 sequences from those of the same genotype analyzed in previous years (Supplementary Table S2 to S12).

**Table 3:** Nucleotide identity and divergent of VP1 gene sequences of genotype IV strains from Bhutan compared with that of genotype IV strains from other countries in 2002–2017 and 2023.

Patient	Specimen No	Specimen Type	GenBank accession no	Nucleotide identity		Nucleotide divergent	
				Genotype IV 2002–2017 (n = 46)	Genotype IV 2023 (n = 22)	Genotype IV 2002–2017 (n = 46)	Genotype IV 2023 (n = 22)
Patient #1	RCDC-001C	Conjunctival Swab	PP505845	89.32–94.93	98.78–99.23	5.07–10.62	0.77–1.22
	RCDC-010 T	Throat/Nasal Swab	N/A				
Patient #2	RCDC-002C	Conjunctival Swab	PP505846	89.19–94.81	98.67–99.12	5.19–10.81	0.88–1.33
	RCDC-011 T	Throat/Nasal Swab	N/A				
Patient #3	RCDC-003C	Conjunctival Swab	N/A				
	RCDC-012 T	Throat/Nasal Swab	PP505851	89.18–94.81	98.67–99.12	5.19–10.82	0.88–1.33
Patient #4	RCDC-004C	Conjunctival Swab	PP505847	89.19–94.81	98.67–99.12	5.19–10.81	0.88–1.33
	RCDC-013 T	Throat/Nasal Swab	N/A				
Patient #5	RCDC-005C	Conjunctival Swab	PP505848	89.19–94.81	98.67–99.12	5.19–10.81	0.88–1.33
	RCDC-014 T	Throat/Nasal Swab	PP505852	89.19–94.81	98.67–99.12	5.19–10.81	0.88–1.33
Patient #6	RCDC-006C	Conjunctival Swab	N/A				
	RCDC-015 T	Throat/Nasal Swab	N/A				
Patient #7	RCDC-007C	Conjunctival Swab	N/A				
	RCDC-016 T	Throat/Nasal Swab	N/A				
Patient #8	RCDC-008C	Conjunctival Swab	PP505849	89.19–94.81	98.67–99.12	5.19–10.81	0.88–1.33
	RCDC-017 T	Throat/Nasal Swab	N/A				
Patient #9	RCDC-009C	Conjunctival Swab	PP505850	89.73–95.30	99.12–99.56	4.70–10.27	0.44–0.88
	RCDC-018 T	Throat/Nasal Swab	PP505853	89.73–95.30	99.12–99.56	4.70–10.27	0.44–0.88

## Discussion

Since its first documented outbreak in Ghana and Nigeria in 1969, AHC has been a persistent global health challenge^4^. In Bhutan, annual conjunctivitis cases showed a notable decline from 2014 to 2022, with reported cases decreasing from 32,054 in 2014 to 12,217 in 2022. This trend, documented in the Annual Health Bulletin of Bhutan (2023 edition), suggests effective management of the disease within outpatient settings^21^. Additionally, NEWARSIS recorded only two outbreaks during this period, in 2018 and 2020.

However, the year 2023 marked a deviation from the previous trend, as outlined in the Annual Health Bulletin of Bhutan (2024 edition)^22^. A total of 38,199 cases were reported, accompanied by multiple outbreaks of AHC documented through the NEWARSIS. These outbreaks were characterized by increased severity and transmissibility, contrasting with the self-limiting and localized nature of past occurrences. The outbreak dynamics shifted from predominantly affecting school-aged children to a broader demographic, including adults closely associated with the initial patient group^17,23^. Interestingly, during the COVID-19 pandemic (2020–2022), Bhutan recorded its lowest incidence of conjunctivitis in decades. Annual cases fell from 22,637 in 2018 and 22,233 in 2019 to just 13,131 in 2020, 12,822 in 2021, and 12,217 in 2022^21,22^. This sharp decline is likely attributable to the stringent public health measures implemented nationally, including mask-wearing, enhanced hand hygiene, and social distancing, which significantly reduced the transmission of not only COVID-19 but also other communicable diseases such as viral conjunctivitis. This observation aligns with global patterns where declines in infectious conjunctivitis cases were reported following the adoption of COVID-19-related public health protocols ^24,25^. These trends underscore the potential of broad public health strategies to control not only the targeted infectious disease but also other communicable diseases.

In this study, weak positive results (Ct values > 30) were obtained for HAdV, HBoV, and Influenza A using the FTD21 multiplex PCR assay. However, genome sequences for these viruses were not detected by NGS, despite the inclusion of specific probes for these viral genomes in hybrid-capture target enrichment. Conversely, all five samples with weak positive results for EV and HRV (Ct values > 30) were confirmed through NGS, which identified them as CVA24v genotype IV. This discrepancy highlights potential limitations of the FTD21 assay in this context and suggests that further testing with an independent PCR system is warranted to clarify the results. Specific primers and probes for detection of EV70 and CVA24v were not available for this study. Therefore, we used the FTD21 multiplex real-time RT-PCR assay for an initial assay. While this assay is broad in scope, it may not have been optimized for detecting CVA24v or EV70 (Tube-5 reaction was for detection of RSVA/B, HAdV, EV, and HPeV), potentially contributing to the observed discrepancies. One plausible explanation for the results is that the primers and probes used in the FTD21 assay may not perfectly match the CVA24v genome sequences, leading to reduced amplification efficiency and higher Ct values.

NGS demonstrated greater sensitivity than the FTD21 multiplex PCR assay in detecting CVA24v genotype IV in two samples that were negative by multiplex PCR. This finding supports the value of NGS in outbreak investigations, particularly when existing diagnostic methods may not fully account for the diversity of circulating pathogens. Given that EV70 and CVA24v are well-documented causes of acute hemorrhagic conjunctivitis (AHC), using a more targeted PCR approach, such as an EV-specific assay followed by VP1 sequencing, might have been more effective than the general FTD21 multiplex PCR assay for this investigation. Future studies should consider employing assays optimized for the pathogens most likely to be involved in AHC outbreaks.

Regarding the specimen types, CVA24v was detected more frequently in conjunctival specimens than in throat/nasal swabs (67% versus 33%). However, the lack of information on the day of symptom onset limits the ability to analyze the association between symptom onset and positivity in different specimen types. Previous studies suggest that early testing of conjunctival specimens (within 2–3 days of symptom onset) enhances enterovirus detection, whereas later testing may benefit from combined oropharyngeal and conjunctival swab collection. This underscores the importance of optimizing sampling strategies for accurate diagnosis^9,15^.

The CVA24v genotype IV has been characterized by extremely rapid worldwide spread after 2000 and due to this, has been previously reported to have emerged as a predominant strain across the globe^13^. Reemergence of this genotype in 2023 was reported in AHC outbreaks in northern India (July to August) and Pakistan (September)^15,16^. In addition, information from GenBank indicated that CVA24v genotype IV sequences from China were also identified in May to June 2023; however, no relevant reports were available at the time of writing this manuscript. The date collection of these sequences suggests that virus circulated and caused outbreaks in China (May to June 2023), India (July to August 2023), Bhutan (June to October 2023), and Pakistan (September 2023). Phylogenetic trees from our study highlight that the new CVA24v genotype IV strains that reemerged in 2023 are clustered in a separate group from the viruses of the same genotype that circulated in previous years (2002–2017). The novel mutations identified in the 2023 CVA24v genotype IV sequences may be contributing factors to the reemergence and high transmission rate of the disease, which has contributed significant outbreaks in China, India, Bhutan, and Pakistan within a short time frame (May to October 2023). Unfortunately, the association of these mutations with disease severity cannot be analyzed due to the limitations of the analysis and the need for further clinical data to fully understand the implications of the identified mutations.

In summary, our paper sheds light on the recent conjunctivitis outbreak in Bhutan, highlighting the significance of genomic sequencing in monitoring and understanding the transmission of contagious illnesses. The significant genetic similarity observed between Bhutan and other countries underscores the need for public health measures to mitigate the spread of conjunctivitis. This finding not only emphasizes the importance of continued surveillance and monitoring but also lays the groundwork for future outbreak response strategies and the development of targeted interventions to prevent and control AHC in Bhutan. Subsequent investigations should prioritize unravelling the functional consequences of the detected mutations and devising approaches for preventing and managing future outbreaks. In addition, the successful collaboration between the RCDC Bhutan and WRAIR-AFRIMS highlights the importance and effectiveness of international cooperation in addressing public health challenges, demonstrating the value of sharing resources and expertise to enhance disease surveillance and response efforts.

Our study has certain limitations. The lack of individual case-based clinical data limits the ability to fully assess the implications of identified mutations. Additionally, laboratory analyses were conducted on a limited number of samples, all collected from Phuntsholing, potentially limiting geographic representation. Future studies should include a broader geographic sample to provide a more comprehensive understanding of AHC outbreaks in Bhutan.

## Methodology

### Ethics statement

The specimens were collected as a part of disease outbreak investigation mandated by the Disease Outbreak Investigation and Control Manual V1 2015 and NEWARS guideline (www.rcdc.gov.bt/web/). Hence, no written consent was obtained from patients. To further characterize the specimens, the RCDC provided a written request for public health support to the WRAIR-AFRIMS, Bangkok, Thailand for analysis of de-identified specimens by RT-PCR and Next Generation Sequencing (NGS). The material was reviewed by the Walter Reed Army Institute of Research (WRAIR) and clearance was obtained for its presentation and/or publication (WRAIR No. 3105).

### Outbreak notification

Bhutan established the Notifiable Disease Surveillance System (NDSS) in 2010, which was later revised into the NEWARS system. This comprehensive, web-based national surveillance and response system monitors priority diseases and syndromes of public health concern. NEWARS integrates indicator-based surveillance (IBS) for 11 weekly and 15 immediately notifiable diseases and event-based surveillance (EBS) for ad hoc reporting of unusual health events.

During the AHC outbreak in 2023, the EBS component was used to collect outbreak information by reporting clusters of AHC cases. AHC cases are defined as individuals presenting with acute bilateral or unilateral redness of the eye(s), pain or discomfort, and watery discharge, with no other underlying cause identified. A risk assessment tool, outlined in the NEWARS guideline, evaluated epidemiological factors such as case numbers, potential for widespread outbreaks, morbidity, mortality, and contamination risks based on eight pre-set questions. Each question was scored, and the system classified the outbreak risk as low, medium, or high. Based on the risk level, RCDC provided recommendations to reporting healthcare centers. Local reporting sites submitted investigation reports with basic epidemiological data, including the number and characteristics of cases, clinical manifestations, geographical distribution, and control measures implemented. In addition, RCDC increased outreach to other health centers to increase reporting of similar events in their localities and as of this report, outbreaks of AHC continue to be tracked through NEWARS surveillance, particularly from southern bordering districts with India and within central Bhutan. Additionally in hospitals with ophthalmologists, RCDC requested to ship samples for laboratory investigation as part of outbreak response.

### Specimen collection, Storage and Shipment

Eighteen paired samples (9 conjunctival and 9 throat/nasal swabs) were collected from 9 patients on 17 August 2023, in Phuntsholing. This selection was part of an exploratory investigation to identify a potential etiological agent during a large-scale outbreak of AHC. The outbreak disproportionately affected vulnerable groups, such as school children, and was characterized by similar clinical symptoms among reported cases. The decision to collect a limited number of samples was due to Bhutan’s constrained diagnostic capacity for identifying the causative agent of AHC. Given these limitations, we focused on a small, representative subset to maximize the likelihood of detecting the pathogen while conserving resources.

Specimens were collected in Universal Transport Medium (UTM), provided by Copan, California, USA, and subsequently shipped to RCDC. The specimens were transported in insulated containers with ice packs to ensure that the temperature remained between 2–8 °C throughout transit. Temperature storage conditions were monitored to prevent degradation of the samples and preserve their integrity for further analysis. Upon receipt, the specimens were stored at −80 °C for further investigation. Specimens were initially tested for influenza virus using the FLU real-time RT-PCR assay following the CDC protocol. Influenza-negative specimens were subsequently shipped on dry ice to WRAIR-AFRIMS for further analysis. At WRAIR-AFRIMS, total nucleic acids were extracted and analyzed using the FTD21 multiplex real-time PCR assay for the preliminary detection of pathogens. Next-generation sequencing (NGS) was performed to confirm the presence of pathogens identified by the FTD21 assay; however, only 17 specimens underwent NGS due to insufficient volume in one specimen.

### Nucleic acid extraction

Total nucleic acid (NA) was extracted from 200 µl of UTM from collected 18 specimens, which contained a conjunctival or throat/nasal swab, using the MagNA Pure 96 DNA and Viral NA Small Volume Kit (Catalogue No. 06543588001, Roche Life Science, Switzerland), on MagNa Pure 96 instrument (Roche Life Science, Switzerland), following the manufacturer’s instructions. The elution buffer was automatically added to archieve a final NA volume of 50 µl.

### Multiplex real-time RT-PCR

A total of 50 µl of extracted total NA was used for multiplex real-time RT-PCR testing with the Fast-Track Respiratory 21 (FTD21) assay (Catalogue No. SMN 10,921,703, Siemens Healthineers, Berkeley, CA) following the manufacturer’s instructions. The assay utilized equine arteritis virus (EAV) as an internal control (IC), which was added to each sample and the negative control during the extraction process. The IC was extracted, processed, and amplified simultaneously with each sample to monitor the extraction process and identify any PCR inhibition. The FTD21 assay uses five PCR reaction tubes, each containing a proprietary mixture of primers and probes for detecting respiratory pathogens. A 10 µl of extracted total NA was added to the reactions in Tube-1 through Tube-5 as a template for pathogens detection. The targets for each tube were as follows: Tube-1 reaction was for detection of influenza A (Flu A), influenza A subtype H1N1 (pandemic H1N1), human rhinovirus (HRV), influenza B (Flu B). Tube-2 reaction was for detection of human coronaviruses NL63 (HCoV-NL63), 229E (HCoV 229E), OC43 (HCoV-OC43), and HKU1 (HCoV HKU1). Tube-3 reaction was for detection of human parainfluenza viruses, 2, 3, and 4 (HPIV- 2, 3 and 4 and internal control. Tube-4 reaction was for detection of human parainfluenza viruses-1, Mycoplasma pneumoniae (M.pneu), human bocavirus (HBoV), human metapneumovirus (HMPV A/B). Tube-5 reaction was for detection of respiratory syncytial virus (RSVA/B), HAdV, EV, human parechovirus (HPeV).The real-time RT-PCR was conducted on an ABI7500 Fast PCR instrument (Life Technologies, USA) following the manufacturer’s instructions, using the following thermal cycler program; 50 °C for 15 min, 94 °C for 1 min, 40 cycles of 94 °C for 8 s, 60 °C for 1 min. The cycle threshold (Ct) value was used to determine the presence of pathogens according to the FTD21 assay manual. A Ct value of 40 was considered negative, while a Ct value of less than 33 with a typical S-shaped amplification curve was considered positive. Samples with Ct values between 33 and 40 required repeat testing for confirmation.

### NGS with hybrid capture-based target enrichment

Prior to library preparation, 50 µl of extracted total NA was precipitated by adding 25 µl (0.5 volume) of 7.5 M ammonium acetate and 165 µl (2.2 volume) of ice-cold absolute ethanol. The mixture was centrifuged at 14,000 rpm for 15 min at 4 °C. The supernatant was carefully removed and discarded, ensuring the pellet remained undisturbed. The pellet was washed twice with ice-cold 70% ethanol to remove impurities. After washing, the pellet was air-dried at room temperature for approximately 10 min, or until no residual liquid was visible. Finally, the pellet was resuspended in 8.5 µl of nuclease-free water for subsequent library preparation. The Viral Surveillance Panel kit (VSP, Catalogue No. 20088154, Illumina, USA) was used following the manufacturer’s instructions for library preparation and hybrid-capture target enrichment for detection of 66 viral genomes (listed in supplementary Table S1)^26^. Briefly, 8.5 µl of the resuspended NA sample with concentration ranging from 10 to 100 ng was denatured and used for first and second-strand cDNA syntheses followed by tagmentation and cDNA library construction. DNA libraries were cleaned with Agencourt AMPure XP (Beckman Coulter, USA) before normalization in resuspension buffer. For three-plex enrichment, three libraries were each diluted to a concentration of 200 ng in a volume of 2.5 µl and then combined into one pool. Subsequently, 7.5 µl of the pool was used for target enrichment. Target enrichment was performed by bead-based capture of hybridized probes, amplification, and purification. For quality control check, fragment size of enriched libraries was analyzed using QIAxcel Advanced System (QIAGEN, Germany). Concentration of enriched libraries were quantified using Qubit dsDNA HS Assay Kit (Thermo Fischer Scientific, USA). Enriched libraries were equimolarly pooled at 2 nM and diluted to a loading concentration of 0.8 pM. The pool was then sequenced (15 samples per run) on a MiSeq instrument using a 500-cycle (2 × 250-bp, paired-end) MiSeq v2 reagent kit (Illumina), following the manufacturer’s instructions.

### NGS data analysis

The obtained paired-end reads were subjected to quality trimming using BBtools v.37.62 with the following criteria: base quality threshold of > 25, minimum length requirement of 100, and minimum average quality threshold of 20^27^. The viral genome consensus sequence of each sample was derived by aligning the trimmed reads to the CA24v reference genome sequence (KR478685.1) from the US National Institute of Health (NIH) GenBank nucleotide database. This alignment was performed using BWA-MEM v.0.7.17^28^ and iVAR v1.3.1^29^ with the following criteria: mapping quality threshold > 30, base quality > 30, and a minimum of depth of coverage of 10. Ambiguous bases were identified and confirmed by genome-guided assembly using Trinity v2.14.0^30^. The obtained consensus sequences were aligned with other sequences in the NCBI database to search for sequence similarity using BLAST^31^.

Phylogenetic analysis was conducted using maximum-likelihood (ML) trees constructed for the VP1 gene (915 nucleotides) and the complete coding sequences (CDS, 6,645 nucleotides) of CVA24v. The analysis included sequences generated in this study along with additional sequences retrieved from GenBank. From a dataset of 1,620 CVA24v sequences available in GenBank as of November 2023, a random subset of 96 VP1 gene sequences and 83 CDS sequences were selected for ML tree construction. Nucleotide alignment was performed using MAFFT v7.31019, and tree building using IQ-TREE v2.0.3^32,33^. The trees were constructed with 1,000 ultrafast bootstrap replicates and visualized using FigTree v1.4.4^34^. The Treesub program (https://github.com/tamuri/treesub) was utilized to deduce amino acid substitution along the tree. MEGA11 software was used to identify amino acid substitutions^35^. Molecular CVA24v genotyping based on the complete genome sequences was also performed using online Genome Detective, Enterovirus Genotyping Tool v.2.1321^36^.

## Supplementary Information

Supplementary Information 1.

Supplementary Information 2.

## Data Availability

Eight complete CVA24v genome sequences and one partial genome obtained in this study were deposited in the NCBI GenBank database under accession number PP505845-PP505853.
